# Prefrontal Cortex Hemodynamics During Exercise in Older Adults With Motoric Cognitive Risk Syndrome

**DOI:** 10.1093/geroni/igaa057.612

**Published:** 2020-12-16

**Authors:** Kell Grandjean da Costa, Eduardo Fontes, Alekya Menta, Sarah White, Roger Fielding, Christopher Kowaleski, Nathan Ward, Kieran Reid

**Affiliations:** 1 Tufts University, Medford, Massachusetts, United States; 2 Tufts University, Medford, United States; 3 Tufts University, Boston, United States; 4 Tufts University, Boston, Massachusetts, United States; 5 Somerville Council on Aging, Somerville, Massachusetts, United States

## Abstract

The motoric cognitive risk syndrome (MCR) is a recently described pre-dementia syndrome in older adults characterized by slow gait coupled with subjective cognitive complaints. While previous studies have demonstrated the benefits of exercise on cerebral hemodynamics in healthy older adults, to date, no study has characterized the effects of exercise on these parameters among more vulnerable older persons with MCR. To address this knowledge gap, we investigated how the brain area responsible for high-order cognitive function (i.e., prefrontal cortex) is affected during acute cycling exercise in 19 older adults with MCR (Age (mean ± SD): 73.7 ± 7.1 years; BMI: 32.1 ± 5.5 kg/m2; gait speed: 0.55 ± 0.1 m/s; Modified Mini-Mental score: 91.8 ± 6.8; 74% female). Participants performed an incremental submaximal cycling test and we used functional near-infrared spectroscopy to assess changes in concentrations of Oxyhemoglobin (O2Hb), Deoxyhemoglobin (dHb) and total hemoglobin (Hbt) during exercise. Results showed that participants cycled for 4.9 ± 0.5 minutes, achieved a submaximal load of 54.7 ± 17.3 watts, a peak exercise heart rate of 95.7 ± 14.7 beats/min and a rate of perceived exertion (13.8 ± 2.0). Compared to baseline, there was an increase of 97.3 % in the O2Hb, 86 % on the Hbt and an 87.9 decrease of dHb while exercising. Our findings suggest that acute exercise at light through moderate intensity increases prefrontal cortex oxygenation in older adults with MCR. Additional studies are also warranted to characterize the effects of chronic exercise on cerebral hemodynamics in at-risk older adults.

